# Welcome to the new editorial team

**DOI:** 10.1186/s13047-018-0298-6

**Published:** 2018-10-19

**Authors:** Keith Rome, Catherine Bowen

**Affiliations:** Auckland University of Technology, Auckland, New Zealand

It is a great honour and privilege to be appointed for the new editorial team of the *Journal of Foot and Ankle Research* (JFAR). We would like to thank our predecessors Professor Hylton Menz, Professor Alan Borthwick and Mike Potter for all the hard work over the last 10 years. During this time they have served the Podiatric Community with dedication, commitment, and diligence while bringing to the journal a strong vision and a wealth of knowledge that has tremendously improved the visibility and impact of the journal. Our three predecessors have been recognised for their work as Emeritus Editors for the journal. The new editorial team will continue to seek their expert guidance for taking JFAR to further heights of recognition and readership.

Professor Bowen and I have had the privilege of serving as Deputy Editor and Associate Editor, respectively for JFAR. Therefore, we understand the nuances and importance of peer review in the manuscript handling process. We believe that scientific values must be upheld in manuscripts and that the editorial team should work efficiently. The editorial team will be strengthened by new Deputy Editors (Andrew Buldt, Michelle Spruce) and Associate Editors (Cylie Williams, Anita Williams and Daniel Bonanno), as well as members of the editorial board and advisors from all over the globe.

We believe that the most critical components of any scientific journal’s success are the submission of high-quality manuscripts, the dedication of members of its Editorial Board, and excellence of those reviewing the manuscripts. These days, when publication in major journals can take several months after a paper is accepted, the open-access online format of JFAR provides a platform for fast publication without undue delay.

Looking back over its last 10 years, the journal has published on a wide diversity of research. Perhaps the most rewarding aspect of the journal has been the opportunity provided to first-time authors to have their work published. There is a bias in academic publishing towards those experienced academics with established reputations, and it is vital that early career scholars are given the prospect of publishing their work. Any academic journal is by definition a collaborative activity, involving the energy and goodwill (and often the patience!) of authors, reviewers, editorial team and production staff. Another lesson we learn from innovation is that it is a continuing process, and change is inevitable and to be welcomed.

As so many of the articles in this journal argue, innovation is a long-term concern, and assessing its achievements requires a long-term perspective. Our vision would be initially to continue with the current format but in the future consider broadening the scope of the journal by adapting ‘new emerging themes’ through proactively seeking and promoting thematic issues. This future vision will need to be endorsed by Australian Podiatric Association, College of Podiatry (UK) and ably supported by our allies in the Canadian Federation of Podiatric Medicine. Finally, we would like to thank at this very early stage all those involved in building the journal over the years, and especially all the authors and referees for all their hard work in enhancing scholarship. The journal is now well established. It has a first-rate publisher in BMC; with excellent distribution, and is included in ISI Web of Science. The number and quality of the submissions continues to improve from its already strong base.

**Professor Catherine Bowen and Professor Keith Rome**

**Editors-in-Chief**

